# The Moderating Effects of Social Responsibility Climate and Safety Climate in Keeping Healthcare Workers’ Engagement during COVID-19

**DOI:** 10.3390/healthcare11081077

**Published:** 2023-04-10

**Authors:** Bin Ding, Tianyi Miao

**Affiliations:** International Business School Suzhou, Xi’an Jiaotong-Liverpool University, Suzhou 215123, China

**Keywords:** healthcare workers, nurses, social responsibility climate, safety climate, work engagement, COVID-19

## Abstract

Objective: The outbreak of COVID-19 brings an overload of physical and mental demands to healthcare professionals. Keeping healthcare professionals sustainable, engaged, and performing at their highest levels becomes critical and nonetheless difficult. The objective of this research is to link the literature on organizational climates, corporate social responsibility, safety science, and work engagement, and propose a research framework that investigates the factors influencing healthcare professionals’ engagement during COVID-19. Methodology: We propose that when healthcare workers’ career callings are triggered by COVID-19, it influences their perceptions of the work’s meaningfulness, which ultimately enhances their work engagement. We argue that creating a social responsibility climate and a safety climate inside the hospital facilitates the process of turning healthcare workers’ perceived work meaningfulness into work engagement. We collected data from 112 healthcare professionals, including nurses, doctors, and executive staff, from 16 wards in a public hospital in China to test our hypotheses. Results: Hierarchical linear regression analysis provided empirical support for our research model. We find that healthcare professionals’ career callings during COVID-19 enhanced their perceived work meaningfulness, which results in increased work engagement. Moreover, a social responsibility climate and a safety climate strengthens the link between work meaningfulness and work engagement among participants. Conclusions: Creating a social responsibility climate and a safety climate in the workplace are effective management approaches to realize healthcare workers’ feelings of work meaningfulness and turn them into work engagement.

## 1. Introduction

The COVID-19 pandemic created a global health crisis. After the outbreak of COVID-19, healthcare workers played a critical role in delivering timely healthcare service and around-the-clock treatment. Not only are they the ones who are racing against the clock and fighting at the frontline, but the whole profession is feeling a pull in the heart to help. The special period of the COVID-19 pandemic, on the one hand, incites healthcare workers’ sense of occupational calling and professional accountability; on the other hand, it forces them to engage themselves and perform under overloaded physical and mental demands [1]. Keeping healthcare workers sustainably engaged and performing their professional skills at the highest levels becomes critical and nonetheless difficult. Sustainable Human Resources Management (SHRM) research indicates that a series of human resource activities and practices may help organizations to achieve sustainable development goals and sustainable business outcomes [2]. Following this line of thought, in order to keep healthcare professionals’ work engagement sustainable under high work pace and demanding jobs during COVID-19, according to corporate operations, processes and deployment should be employed. Past research indicates that the organizational climate, which refers to the shared perceptions of employees regarding an organization’s policies, procedures, and practices, as well as the types of behavior that are rewarded and supported in work settings [3], plays a critical role in enhancing the organizational sustainability of enterprises. Furthermore, an organizational climate keeps humans thriving and sustainable at the individual level and influences the relational sustainability between team members [4,5,6]. In the current research, an organizational climate is proposed to be a strategic corporate operation that may help to keep healthcare workers sustainably engaged. We consider a social responsibility climate as well as a safety climate to be important during a crisis such as COVID-19. We choose these two organizational climates because a social responsibility climate indicates to employees the socially responsible actions they should take and what they should do for the benefit of the greater good [3,4] while a safety climate ensures that healthcare workers’ safety is a priority during treatment which could be perceived as background support during a crisis [4,6]. 

Past research indicates that during a crisis, people’s career calling may fluctuate during a short period of time [7,8]. We think the starting of COVID-19 offers good timing for studying healthcare workers’ career callings. Career calling, or occupational calling, is defined as “a transcending passion to use one’s talent and competencies toward a positive societal impact and a sense of meaningfulness is derived from the work in a chosen occupational domain” [1], and it becomes even more salient during the situation of COVID-19. Due to the severity of the pandemic, healthcare workers became warriors in society, and the huge amount of societal and professional demands greatly triggered an inherent career calling among them and urged them to focus and engage in their work [1,9]. This research aims to explore how healthcare professionals translate their career calling into work meaningfulness and then work engagement—a state where nurses connect with their work physically, cognitively, and emotionally [10]—during COVID-19. Furthermore, this research aims to demonstrate that building and maintaining a social responsibility climate and a safety climate in the workplace are important organizational operations to translate healthcare professionals’ work meaningfulness into work engagement. 

### 1.1. Calling, Meaningfulness, and Work Engagement

Scholars theorize that a tripartite model of work orientation is such that people tend to see their work as a job, a career, or a calling [8,11]. People who tend to see their work as a job focus on the material benefits; people with a career orientation work for promotion and advancement. When people see their work as a calling, they work for the fulfillment brought by the work [12]. A severe situation of crisis, such as COVID-19, is able to incite nurses’ career calling [1]. When society is facing a crisis, it normally comes along with strained medical staff and strained healthcare professional services. The scarce medical resources and societal needs make nurses realize that their work is salient and in urgent need, so nurses tend to feel a pull in their hearts and feel fortunate to help meet those demands, and hence, tend to see their work as a calling [9]. When people see their work as a calling, they believe that their work contributes to the greater good and makes the world a better place [12]. In this case, during crisis, when healthcare workers feel a calling, they experience spiritual significance, personal fulfillment, and worldly impact, and hence, they are more likely to find their work meaningful [13,14,15,16].

Work meaningfulness refers to the amount of significance people perceive in their work [12]. Healthcare workers’ jobs are widely perceived as meaningful but challenging, especially when confronting a worldly healthcare crisis, such as COVID-19. Past research indicates a link between career calling and work meaningfulness, which is particularly salient when the work is perceived as challenging. A severe crisis signals urgent societal needs, and nurses have strong feelings of obligation and responsibility to fully utilize their professional skills [17]. For example, Dobrow and colleagues in longitudinal research show that people with strong early career callings were more likely to pursue challenging careers professionally in adulthood [18]. We thus propose that during COVID-19, career calling is positively related to perceived work meaningfulness among healthcare workers.

Work meaningfulness is theorized to invoke people’s deeper consideration of purpose and significance [12]. Employees who experience psychological meaningfulness feel that they are receiving a return on investment in a currency of physical, cognitive, or emotional energy. Work meaningfulness may serve as a psychological driving force that makes nurses more energized, activated, and engaged in their work, and that is a high level of work engagement [19,20]. Past research shows that work meaningfulness influences important work outcomes, including work engagement, commitment, performance, job satisfaction, life satisfaction, and general health states [21]. We focus on healthcare professionals’ engagement because during COVID-19, due to the heavy workload, fast work pace, and high physical and mental demands, it was very often that healthcare professionals felt burnt out, depressed, or intended to quit [22]. When healthcare professionals find meaning in their work, they have a stronger intention to continue working as nurses [23]. Following this line of thought, we think that healthcare professionals who find their work meaningful not only would like to continue working as nurses, they are more likely to engage highly in their work. Past research shows that work meaningfulness invokes some of the most important outcomes in organizational studies, including work engagement [12]. We propose that this relationship is particularly salient among healthcare workers during COVID-19. Therefore, we propose the following two hypotheses:

**Hypothesis** **1** **(H1).** *Calling is positively related to work meaningfulness.*

**Hypothesis** **2** **(H2).** *Work meaningfulness is positively related to work engagement.*

### 1.2. Social Responsibility Climate and Safety Climate as Moderators

When healthcare workers are fighting at the frontline, they run the risk of being exposed to the virus and infected individuals, especially during a severe situation, such as COVID-19 [1]. According to a recent report, due to their exposure, a healthcare worker presented as one of the first reinfections reported in the world [24]. On the one hand, a situation of crisis brings about a high level of societal demands so nurses feel a sense of responsibility to assist with intensive caring needs; on the other hand, they feel worried because they have a high risk of being infected. Such worries and negative psychological states sometimes make healthcare workers distracted and hard to keep engaged. From a sustainable human resources management (HRM) perspective, we consider building a social responsibility climate and a safety climate in the workplace, which is a salient sustainable practice that potentially boosts nurses’ feelings of work meaningfulness, which strengthens their engagement and helps to strengthen the effect. 

A social responsibility climate refers to “employees’ shared perceptions concerning organizational stakeholders’ values, expectations, and practices that emphasize the responsibility of individuals as a member in society” [25] (p. 667). Stakeholders of hospitals, especially in a situation of crisis, include wide groups of people, such as the hospital, co-workers, current patients, potential patients, the healthcare system, and society [26,27]. Healthcare workers’ feelings of social responsibility are discretionary and not explicitly recognized by the formal human resource systems [25]. In a unit with a social responsibility climate, healthcare workers are triggered to contribute because they feel the responsibility to help and to relieve the intensive societal needs [28]. Even though they are sometimes exhausted and depressed, a feeling of social responsibility may buffer their negative psychological states and pull them to engage. In this way, regarding nurses who perceive their job as significant to society (i.e., work meaningfulness) and would like to make the best use of their professional skills and capabilities to contribute, when they perceive a social responsibility climate in the workplace, they feel even more energized. We thus propose that a social responsibility climate boosts the effect of work meaningfulness on work engagement.

**Figure 1:**
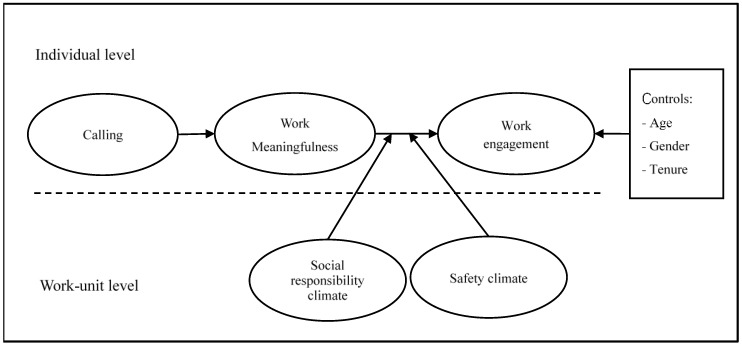
Research model.

The other organizational climate we propose to be contextually important during COVID-19 is a safety climate. A safety climate refers to nurses’ shared perceptions of practices, policies, and procedures as well as the types of behavior that are rewarded and supported regarding the safety of their work environment [29]. COVID-19’s severity and influence never seem to decrease, and it is common that people are nervous and worried that they will be infected by the virus or infected individuals. This is even true for nurses who are frontline warriors and have the closest and most frequent interactions with COVID-19 patients [30]. Long-term worries and negative psychological states can make nurses distracted, depressed, and can make it hard to concentrate. In this case, a safety climate inside working units may work as a buffer, potentially creating a comparatively safe workplace by helping to relieve nurses’ psychological tension of being infected and offering them a sense of security when working. Past research shows that employees working in a safety climate have reduced stress, improved job performance and well-being, and improved work ability [31,32,33]. We thus consider a safety climate to be a key situational factor during the COVID-19 pandemic that can possibly enable nurses to turn their feelings of work meaningfulness into a focused, devoted, and absorbed state, as a safety climate strengthens the effect of work meaningfulness on work engagement. Combined, we propose the following hypotheses. Full research model is shown in Figure 1.

**Hypothesis** **3** **(H3).** *A social responsibility climate moderates the effect of work meaningfulness on work engagement, such that the effect is stronger when a social responsibility climate is high rather than low.*

**Hypothesis** **4** **(H4).** *A safety climate moderates the effect of work meaningfulness on work engagement, such that the effect is stronger when a safety climate is high rather than low.*

## 2. Materials and Methods 

### 2.1. Sample and Procedures

We collected our data from nurses, doctors, and executive staff working in a public general hospital in a city located in Southwest China. The majority of participants were nurses from sixteen different wards across the hospital, including pediatrics, oncology, and clinical laboratory. Considering that the nurses and doctors have very busy hospital routines, we contacted the director of the hospital beforehand to obtain permission for our data collection. 

We used paper-and-pencil questionnaires to collect data. The research protocol was approved by the Ethics Committee of Xi’an Jiaotong-Liverpool University. We included consent forms before the questionnaires and assured participants on the front cover of the questionnaire that their responses would be anonymous and kept confidential. Before we officially started the survey, we invited a friend, who used to be a nurse, to finish a pilot test, ensuring the wording used in the survey and the length of the survey were appropriate.

We initially invited 200 participants from the hospital to our survey. We aimed to invite all possible healthcare professionals in the hospital, but the director was worried that the working routines and efficiency might be affected during this process. After discussion, we agreed to send out 130 copies of questionnaires to participants who worked in the wards with a relatively low work pace. Executive staff in the hospital had more flexible work hours compared to nurses and doctors, so we invited them to our survey as well. The heads of each ward helped us to distribute the questionnaires to the participants. Informed consent was obtained from all subjects involved in the study.

We used a longitudinal design for the data collection. In the first wave, we collected all the variables in the research model except for work engagement. One week later, we invited the participants again and asked them to fill out a second wave with the measure of work engagement. This way, we could examine the effect of work meaningfulness on the sustainability of work engagement. We examined work engagement only one week after the predictors because healthcare professionals have a high work pace and job demands, especially during COVID-19, and things change fast. Past research took a one-year or a three-year gap when measuring the sustainability of engagement, while during COVID-19, it was not feasible and had little meaning [34,35]. After the 2 waves, we collected 112 completed questionnaires, with a response rate of 86.15% (95 nurses, 10 doctors, and 7 executive staff). Among all the participants, 84.82% were females. The mean age of participants was 32.13 years (standard deviation = 7.45). Participants reported having an average hospital tenure of 109.6 months (standard deviation = 101.4) and an average ward tenure of 62.67 months (standard deviation = 68.55). 

### 2.2. Measures

We adopted existing measures for all of the variables in this study. We slightly modified some of the items to reflect the hospital context. The original questionnaire was prepared in English, and then translated into Chinese using standard back translation [36]. Respondents rated the measures on a 5-point Likert scale ranging from 1, “strongly disagree”, to 5, “strongly agree”.

*Calling* was measured by a six-item scale adapted from [25]. A sample item was “The work I do feels like my calling in life”. The Cronbach’s alpha of calling was 0.92.

*Work meaningfulness* was measured with a three-item scale from the second version of the Copenhagen Psychosocial Questionnaire [37]. Examples of the items include “My work is meaningful” and “I feel that the work I am doing is important”. The Cronbach’s alpha of calling was 0.87.

*Social responsibility climate* was measured with a three-item scale from [25]. Examples of the items include “In my opinion, my workplace is socially responsible” and “In my opinion, my workplace is genuinely concerned to improve the well-being of society”. The Cronbach’s alpha of calling was 0.91. We aggregated individual-level data to the ward level, so we justified the aggregation by assessing the intra-class correlation coefficients, *ICC*(1) and *ICC*(2). The *ICC*(1) for a social responsibility climate was 0.12, and the *ICC*(2) was 0.53, indicating that we could aggregate the data.

*Safety climate* was measured with a six-item scale from [38]. Examples of the items include “Health professionals are told when they do not follow good safety practices” and “The safety of health professionals is a high priority with management where I work”. The Cronbach’s alpha of calling was 0.91. The *ICC*(1) for safety climate was 0.11, and the *ICC*(2) was 0.49.

*Work engagement* was measured using the Utrecht Work Engagement Scale (UWES) developed in [39]. This measure included three subscales of work engagement: vigor, dedication, and absorption. Vigor was assessed with six items (e.g., “When I get up in the morning, I feel like going to work”). Dedication was measured with five items (e.g., “I am enthusiastic about my work”). Absorption was assessed with six items (e.g., “Time flies when I am working”). Respondents rated work engagement on a 5-point Likert scale ranging from 1, “never”, to 5, “always”. Cronbach’s alpha of work engagement was 0.93.

*Control variables*. Considering that age, gender, and tenure in hospital could have an influence on health professionals’ work engagement, and past research indicated that these demographics may influence employees’ work engagement (e.g., [40,41]), we included participants’ age, gender, and organizational tenure as control variables. Age and organizational tenure were continuous variables. Gender was a dummy variable, where 0 represented female and 1 represented male. 

### 2.3. Analytic Strategy

Our research model is multilevel in nature with participants nested in wards and because calling, work meaningfulness, and work engagement are individual-level constructs. A social responsibility climate and safety climate are ward-level constructs, so we conducted hierarchical linear modeling (HLM) analyses to test our hypotheses. HLM explicitly takes into account the nested data structure and simultaneously estimates the impact of factors at different levels and on individual-level outcomes. 

## 3. Results

### 3.1. Descriptive Statistics

Table 1 presents the characteristics of participants. Table 2 presents the means, standard deviations, and correlations among the variables. Internal consistency reliabilities are presented on the diagonal. 

### 3.2. Hierarchical Linear Modeling Results

Before we performed the HLM analysis, we tested the normality of all the variables in the model. The chi-squares of the Skewness-Kurtosis tests of work engagement, calling, work meaningfulness, social responsibility climate, and safety climate were 0.44 (*p* > 0.05), 2.76 (*p* > 0.05), 6.13 (*p* < 0.05), 3.48 (*p* > 0.05), and 0.10 (*p* > 0.05), indicating that almost all the variables are normally distributed. We performed these tests using the sktest in Stata 14.0.

To perform the HLM, we first entered all the control variables and the predictor, calling, into the model as shown in Model 1 in Table 3. Then, we entered work meaningfulness, social responsibility climate, and their cross-level interactions in Model 2 and Model 3. Lastly, we entered safety climate and its cross-level interactions with work meaningfulness in Model 4 and Model 5. Table 3 presents the analytical results. 

Hypothesis 1 predicted that calling is positively related to work meaningfulness. Ordinary least square regression results, as shown in Model 1 in Table 3, revealed that calling was positively related to work meaningfulness (*b* = 0.49, *p* = 0.000). Therefore, Hypothesis 1 was supported. 

Hypothesis 2 proposed that work meaningfulness is positively related to work engagement. HLM results shown in Model 2 in Table 3 revealed that work meaningfulness was positively related to work engagement (*b* = 0.31, *p* = 0.000). Therefore, Hypothesis 2 was supported.

**Figure 2:**
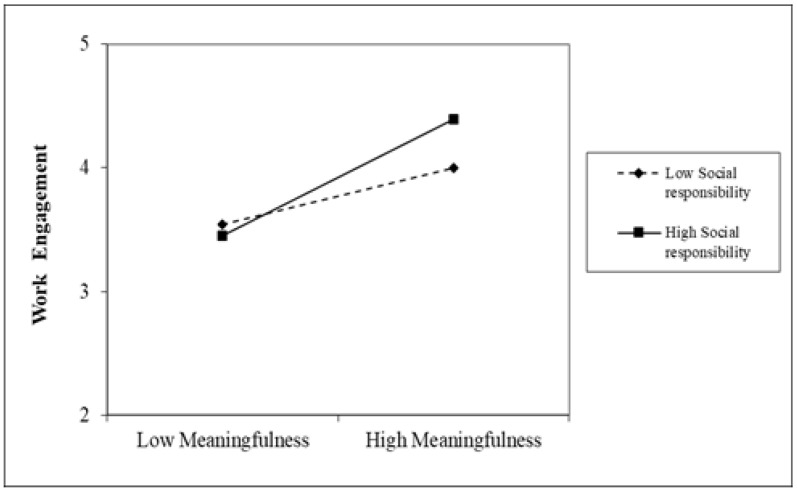
Simple slopes for the interaction between work meaningfulness and social responsibility climate on work engagement.

Hypothesis 3 proposed that a social responsibility climate moderates the effect of work meaningfulness on work engagement, such that the effect is stronger when a social responsibility climate is high rather than low. The HLM results in Model 3 demonstrate a significant interaction between work meaningfulness and a social responsibility climate (*b* = 0.13, *p* = 0.03). We probed the interaction between work meaningfulness and the social responsibility climate by taking one standard deviation above and below the mean to indicate high and low levels. As shown in Figure 2, the simple slopes revealed that the effect of work meaningfulness on work engagement was stronger when a social responsibility climate was high (*r* = 0.44, *p* = 0.000) rather than low (*r* = 0.23, *p* = 0.000). Therefore, Hypothesis 3 was supported.

**Figure 3:**
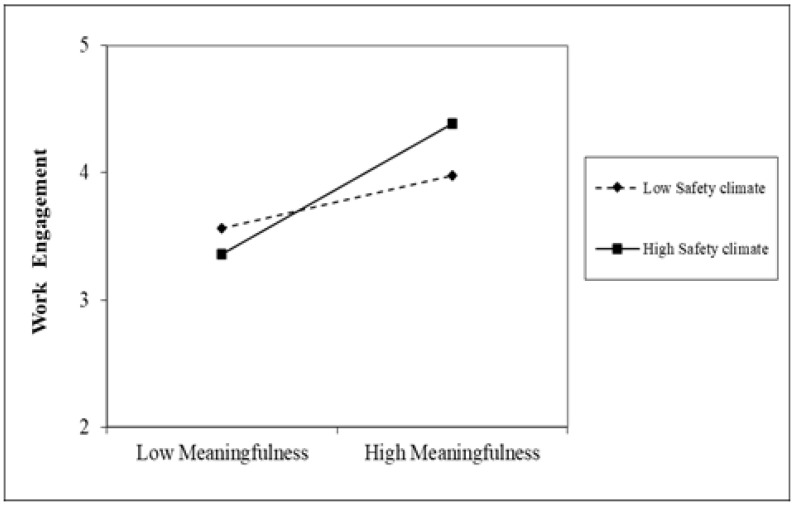
Simple slopes for the interaction between work meaningfulness and safety climate on work engagement.

Hypothesis 4 proposed that a safety climate moderates the effect of work meaningfulness on work engagement, such that the effect is stronger when a safety climate is high rather than low. The results in Model 5 demonstrate a significant interaction between work meaningfulness and a safety climate (*b* = 0.15, *p* = 0.01). We probed the interaction between work meaningfulness and safety climate by taking one standard deviation above and below the mean to indicate high and low levels. As shown in Figure 2 and Figure 3, the simple slopes indicated that the effect of work meaningfulness on work engagement was stronger when the safety climate was high (*r* = 0.48, *p* = 0.000) rather than low (*r* = 0.21, *p* = 0.000). Therefore, Hypothesis 4 was supported.

## 4. Discussion

This research links the literature on organizational climate, corporate social responsibility, safety science, and work engagement, and examines a research framework that aims to keep health professionals’ work engagement sustainable during COVID-19. We find that during COVID-19, the societal and professional demands triggered health professionals’ feelings of career calling, and such callings made them have an enhanced feeling of work meaningfulness—they are helping, they are saving lives, and they are contributing to the greater good—which results in enhanced engagement. Our results further show that during this special period, a social responsibility climate and safety climate serve as situational boosters that strengthen the above link and enable corporations to achieve sustainable engagement among healthcare workers. Below, we discuss the theoretical and practical implications of our findings. 

### 4.1. Theoretical and Practical Implications 

This study makes contributions to the existing literature in the following ways. First, in hoping that the turning period of COVID-19 would arrive soon, healthcare professionals were confronted with overloaded demands that require both physical and mental engagement at a high level [1]. Even so, keeping health professionals sustainably engaged receives limited research attention [34]. Grounded in the broad literature of sustainable HRM, this research seeks to uncover mechanisms that help to achieve the sustainable engagement of healthcare professionals [42]. As shown in Table 3, we find that when a sense of calling is invoked during COVID-19, it positively influences healthcare professionals’ feelings of work meaningfulness, which in turn leads to work engagement. We link research on organizational climates, which reflects the practices, procedures, and policies in an organization, with sustainable HRM research and bridge these two bodies of literature in the context of COVID-19. Our results in Table 3 show that a social responsibility climate and safety climate strengthen the effect of work meaningfulness on work engagement. We demonstrate that building a social responsibility climate and safety climate are indeed effective practices and can be utilized as management strategies to maintain sustainable performance and the engagement of healthcare professionals (as shown in Figure 2 and Figure 3).

Second, this research highlights the importance of organizations’ practices in creating a socially responsible and safe workplace in hospitals. As COVID-19 brings a fast work pace, overloaded professional demands, and unbearable physical and psychological pressure [43], a socially responsible and safe workplace to some extent relieves the pressure, calms the workers, and offers inner motivation to overcome the difficulties. Our results indicate that when healthcare workers find their jobs meaningful and would like to make a contribution, a socially responsible and safe working environment would help to keep them sustainably engaged and potentially realize their eagerness to contribute. 

Third, past research shows that COVID-19 incites high levels of career calling from healthcare professionals [44,45]. This research demonstrates that it is indeed true when a whole society depends on this important group of people. This research further shows that depending only on the inner calling of healthcare workers is not enough to achieve sustainable outcomes because a career calling is not stable, and it fluctuates due to high demands, work pace, and pressure [46]. Our results indicate that hospitals and managers in hospitals should make efforts to trigger this inner calling and translate the inner calling into important work outcomes through policy making [46]. Our results in Table 3 and Figure 2 and Figure 3 show that creating effective organizational climates with regard to social responsibility and safety would be such an important effort. 

This study offers significant practical implications for management. Our results demonstrate the significant impact of calling on the perception of work meaningfulness and sustainable engagement among healthcare workers. In order to realize such an important link, this research indicates that hospitals should endeavor to emphasize a social responsibility climate to healthcare workers and try to make effective policies and practices with regard to safety in hospitals to offer a feeling of security and safety to healthcare workers. These are important management practices because the severity and scope of COVID-19 are huge and broad, and internal supporting practices from the hospitals are key to maintain and promote inner motivation for frontline healthcare workers [44,45,46]. 

### 4.2. Limitations

We are aware of the limitations of this study. First, we collected our second wave data only one week after the first wave to measure the sustainability of work engagement, which was much shorter than previous studies (e.g., [34,44]). Although COVID-19 was a special time, future research could use a longer lag to test the sustainability of healthcare workers’ engagement. Second, we collected all of the variables from one source—healthcare professionals— and although there was a time lag between predictors and outcomes, it is hard for us to draw causal inferences. Third, the results of this study lack external validity given its particularity in the timing of data collection (the outbreak of COVID-19 in China) and location (not far from Wuhan). We, therefore, should be careful if we would like to draw conclusions from the results in a different context.

## 5. Conclusions

Grounded in the broad literature of sustainable HRM, this research links the literature on organizational climate, corporate social responsibility, safety science, career calling, and work engagement. Situated in the context of COVID-19, this research investigates the relationship between healthcare professionals’ career calling, work meaningfulness, and work engagement. The results demonstrate that creating a social responsibility climate and safety climate in the workplace are effective management approaches to realize healthcare workers’ feelings of work meaningfulness and turn them into work engagement. The present research indicates the importance of creating a socially responsible and secure workplace for healthcare workers, especially during COVID-19.

## Figures and Tables

**Table 1:** Subject characteristics.

Variables	Category	Number	Frequency (%)
Gender	Female	95	84.82
	Male	17	15.18
Age	20–30	39	34.82
	31–40	60	53.57
	41–50	13	11.61
Tenure	<24 months	23	20.54
	24–60 months	17	15.18
	60–120 months	35	31.25
	120–240 months	21	18.75
	>240 months	16	14.29
Occupational group	Nurse	93	83.04
	Doctor	14	12.50
	Executive staff	5	4.46

Notes: *N* = 112.

**Table 2:** Means, standard deviations, correlations, and internal consistency reliability.

	Mean	s.d.	1	2	3	4	5	6	7
1. Calling	3.69	0.81	0.927						
2. Work meaningfulness	4.25	0.63	0.646 (0.000)	0.873					
3. Social responsibility climate	4.24	0.63	0.425 (0.000)	0.455 (0.000)	0.911				
4. Safety climate	4.11	0.54	0.563 (0.000)	0.606 (0.000)	0.710 (0.000)	0.905			
5. Work engagement	3.87	0.68	0.866 (0.000)	0.567 (0.000)	0.437 (0.000)	0.561 (0.000)	0.946		
6. Age	32.13	6.29	0.026 (0.788)	0.041 (0.671)	0.041 (0.669)	−0.005 (0.962)	−0.009 (0.926)	-	
7. Gender	0.15	0.36	0.175 (0.065)	0.216 (0.022)	0.296 (0.001)	0.269 (0.004)	0.151 (0.112)	−0.176 (0.063)	-
8. Tenure	109.60	98.62	−0.178 (0.061)	−0.123 (0.196)	−0.195 (0.039)	−0.280 (0.003)	−0.177 (0.077)	0.615 (0.000)	−0.059 (0.531)

Notes: *N* = 112. Cronbach’s alphas are on the diagonal. *P*-values are shown in parentheses.

**Table 3:** Hierarchical linear modeling results: the effects of work meaningfulness, social responsibility climate, and safety climate on work engagement.

Predictors	Model 1	Model 2	Model 3	Model 4	Model 5
Intercept	2.38 (0.33) ***	3.90 (0.07) ***	3.85 (0.07) ***	3.90 (0.07) ***	3.82 (0.07) ***
Level 1					
Gender	0.18 (0.13)	0.01 (0.05)	0.00 (0.05)	0.02 (0.05)	0.00 (0.05)
Age	0.00 (0.01)	0.01 (0.06)	0.02 (0.06)	0.01 (0.06)	0.03 (0.06)
Tenure	−0.00 (0.00)	−0.09 (0.07)	−0.09 (0.07)	−0.08 (0.07)	−0.09 (0.07)
Calling	0.49 (0.06) ***				
Work meaningfulness		0.31 (0.06) ***	0.36 (0.06) ***	0.29 (0.06) ***	0.36 (0.07) ***
Level 2					
Social responsibility climate		0.17 (0.08) *	0.08 (0.08)		
Safety climate				0.18 (0.08) *	0.05 (0.10)
Cross-level					
Work meaningfulness × social responsibility climate			0.13 (0.08) *		
Work meaningfulness × safety climate					0.15 (0.08) **
Level 1 R square	0.43	0.17	0.17	0.17	0.17
Level 2 R square		0.09	0.09	0.09	0.10
Model deviance		165.20	165.20	163.36	163.36

Notes: Level 1 *N* = 112 employees, level 2 *N* = 16 work units; * *p* < 0.05, ** *p* < 0.01, *** *p* < 0.001.

## Data Availability

Data can be requested from the first author.
